# Supplementary material for: Biocompatible low-voltage electrothermal actuators with biological operational temperature range
Source: Commun Mater. 2025 Aug 5;6(1):174. doi: 10.1038/s43246-025-00893-1 (PMC12325083; doi:10.1038/s43246-025-00893-1)
Supplement: Supplementary file 2 — Supplementary Information [file 43246_2025_893_MOESM2_ESM.pdf]

Supporting information: Towards Muscle Replacement: Biocompatible Low-Voltage Electrothermal Actuators

Adela Slavikova,[1] Benjamin C. Baker,[1*] Marcos Villeda-Hernandez,[1] Annabel Coekin,[1] Julia Kwasniewska,[1] Tim Good[2], Mina Aleemardani,[3] Heidi Snethen,[3] James P. K. Armstrong[3] and Charl F. J. Faul[1*]

[1] School of Chemistry, University of Bristol, Bristol, BS8 1TS, United Kingdom

[2] NIHR Long Term Conditions HealthTech Research Centre, Sheffield Teaching Hospitals NHS Foundation Trust, Sheffield, S10 2JF, United Kingdom

[3] Department of Translational Health Sciences, Bristol Medical School, University of Bristol, Bristol BS1 3NY, United Kingdom

Contents

1 Experimental Methods 2

1.1 Chemicals 2

1.2 Bio35 Synthesis and Optimisation 2

1.3 Actuator preparation 3

1.4 Actuator Molds 3

1.5 Gripper actuator 3

1.6 Valve actuator 3

1.7 Actuation 4

1.8 Measuring Rate of Flow 4

1.9 Characterization 4

1.7.1 Fourier-transform infrared spectroscopy (FTIR) 4

1.7.2 Thermogravimetric analysis 4

1.7.3 Differential Scanning Calorimetry (DSC) 4

1.7.4 Force measuring 4

1.7.5 Testing of cyclability 4

1.7.6 Contact angle measuring 5

1.7.7 Cell culture 5

1.7.8 alamarBlue Assay 5

1.7.9 Confocal fluorescence microscopy 5

2 Results 6

2.1 FTIR analysis 6

2.2 TGA....9
2.3 Gripper actuators....9
2.4 Force measurements....11
2.5 Contact Angle measurements....11
2.6 Cyclic actuation....12
2.7 Additional Actuation Data....13
2.8 Valve additional data....14

1 Experimental Methods

1.1 Chemicals

Chemicals (Merck, UK) were used as received without any purficatioin: poly(bisphenol A – Co – epichlorohydrin) glycidyl end capped, 4,4' diaminodiphenylsulphone, poly(propylene glycol) bis(2-amino propyl ether).

For deionized water a water purifier machine (ELGA PURELAB Ultra GE Genetic MK2, UK) was used. For phosphate buffer solution 800 ml of deionized water, 20.214 g of Sodium Phosphate Dibasic Heptahydrate and 3.394 g of Sodium Phosphate Monobasic Monohydrate was prepared and the solution made up to 1 l with deionized water.

3M Kapton Tape (Fisher Scientific, UK) with a 0.07 mm thickness and 12.5 mm width.

Conductive Thread Madeira HC 12 (Madeira, UK), 100% polyamide/Silver plated <100ohm m^{-1}.

Dragon Skin™ 10 VERY FAST (Smooth-On, USA) was used for preparation of silicon form.

1.2 Bio35 Synthesis and Optimisation

The resin Bio 35 was synthesized in a simple one-pot synthesis using the prepolymer poly(bisphenol A – Co – epichlorohydrin) glycidyl end capped (Epikote™ 828) and two crosslinkers, 4,4' diaminodiphenylsulphone (DDS) and poly(propylene glycol) bis(2-amino propyl ether) (PPG) (see Figure 1). To ensure all epoxy groups were fully crosslinked a 1:1 molar ratio of resin to crosslinkers was used. The ratio of DDS to PPG crosslinkers was varied to alter the properties of the final resin. The exact amounts of all reagents used can be found in **Table S1**. All the reagents were mixed at 36°C for 2 hours in a vial using a magnetic stirrer. After this period, the vial was placed in a vacuum oven for 15 minutes to degas.

Table S1: Exact molar and gram quantities used in the synthesis of various prepared resins.

Percentage of DDS	Epikote™ 828 (mmol)	Epikote™ 828 (g)	DDS (mmol)	DDS (g)	PPG (mmol)	PPG (g)
**22.5**	10.6	3.76	2.38	0.59	8.23	3.29

**25**	10.6	3.76	2.66	0.66	7.95	3.18
**27.5**	10.6	3.76	2.94	0.73	7.70	3.08
**30**	10.6	3.76	3.18	0.79	7.43	2.97
**32.5**	10.6	3.76	3.45	0.86	7.16	2.86
**35**	10.6	3.76	3.71	0.92	6.89	2.76
**37.5**	10.6	3.76	3.98	0.99	6.63	2.65
**40**	10.6	3.76	4.24	1.05	6.36	2.54
**42.5**	10.6	3.76	4.51	1.12	6.09	2.44
**45**	10.6	3.76	4.77	1.18	5.83	2.33

1.3 Actuator preparation

To keep the same sizes of the actuators and ease the manufacturing process, we prepared a negative silicon mold, using commercial polymer Dragon Skin. The negative mold was prepared using a 3D printed resin from an SLA printer. The Kapton Tape (0.07 mm thickness, 12.5 mm width) was placed into the silicon mold, sticky side up. To introduce the joule heater a silver conductive thread was placed onto the Kapton Tape layer arranged into a desired shape. Finally, the synthesized resin was poured on the tape with the thread, and the mold with the actuator was placed into an oven for curing for 24 hours at 55°C.

1.4 Actuator Molds

The design of the negative molds was carried out using Fusion 360 (Autodesk, USA) and subsequently printed using a Mono X 6K SLA 3D printer (Anycubic, CN) with a high transparency resin. After printed, the molds were thoroughly washed with isopropyl alcohol (>99% Sigma-Aldrich, DE) and dried to remove uncured resin. A post-curing process was conducted for 20 seconds in a custom-built UV reactor comprising 4 UV LEDs emitting a wavelength of 365nm (UVA) and a 1W power output each. To prevent the inhibition of the curing process of the silicone rubber, the molds were coated with a polyurethane varnish. Positive molds were then created using Dragon Skin silicone rubber (Smooth-On, USA); the components of Dragon Skin A and B were premixed, poured into the prepared molds, and allowed to cure for 30 minutes.

1.5 Gripper actuator

As one of the proofs of concept, we prepared a gripper. Two of the **Bio35** actuators were joint together, Kapton layer inside, using another Kapton Tape. The voltage was applied (Aim-TTi EL155R Digital Bench Power Supply, UK) to a gripper to grab the paper ball weight (weighing 225.1 mg) and hold it till the voltage was cut off.

1.6 Valve actuator

As a proof-of-concept, we prepared a valve. Two **Bio35** actuators were stuck together at the ends, using another Kapton Tape, to form a sphincter, Kapton Tapes inside. After applying the voltage, the sphincter is opening and releasing the water ("urine") from the syringe ("bladder").

1.7 Actuation

The actuator was clipped to the stand. The source (Aim-TTi EL155R Digital Bench Power Supply, UK) was joined to the silver thread ends by crocodiles and graph paper was used for measuring the displacement. The applied voltages were in the interval from 1 to 5V. Before every measurement, 3 preheating cycles were run.

1.8 Measuring Rate of Flow

To quantify the effectiveness of the sphincter design at restricting urine flow a bespoke setup, shown in **Figure 5**, was designed to simulate urination. A thin tube (Low-density Polyethylene, 6 mm wide) representing the urethra was attached using vacuum thread to a 20ml syringe tube which represented the bladder. The average female urethra is 6mm wide, so tubing as near to 6mm as possible was used. The sphincter was placed around the plastic tube. The bladder was kept constantly full of 20 ml by manually topping up and the water allowed to dissipate under gravity.

1.9 Characterization

1.7.1 Fourier-transform infrared spectroscopy (FTIR)

The FTIR measurements were run on a spectrometer with a universal ATR two modular accessory with a diamond crystal (PerkinElmer Spectrum 100, USA). Sample spectra were recorded at wavenumbers between 450 and 4000 cm^{-1} at a resolution of 4 cm^{-1} with 15 scans.

1.7.2 Thermogravimetric analysis

Thermal stability of the formulations of interest DDS 35% (**Bio35**) and 37.5% was studied using a simultaneous thermal analysis instrument (Netzsch STA 449 F1 Jupiter, Selb, Germany); thermogravimetric analysis (TGA) data was obtained. The cured resins (ca. 10 mg) were added to Al_2O_3 crucibles and subjected to a temperature programme from 30 °C to 800 °C at 5 °C min^{-1}, under Nitrogen atmosphere (50 cm^3 min^{-1}).

1.7.3 Differential Scanning Calorimetry (DSC)

The DSC measurements were taken on a DSC25 (TA instruments, UK), the T_g was then analysed using Trios software as an inflection temperature in the DSC curve. A heating and cooling rate of 10°C min^{-1} was used in a temperature range of -50°C to 100°C. Samples were analysed in T_{zero} aluminium pans with hermetic lids.

1.7.4 Force measuring

The force measurements were conducted using a load cell (RB-Phi-203-100g Micro Load Cell, RoboShop, UK) connected to a data acquisition system (DAQ) (NI 779051-01). The load cell outputs were digitized by the DAQ, enabling accurate retrieval and processing of the data. The DAQ system was interfaced with a computer running MATLAB software, which was used for logging, analysing, and visualizing the data accordingly. The actuator was mounted in an uniaxial support, with one end firmly clipped to the fixed side of the support. The free end of the actuator was positioned and aligned in close proximity to the load cell to ensure accurate force measurements at the point of contact.

1.7.5 Testing of cyclability

A laser (Keyence laser LK-G157, UK) was used to measure 200 cycles. The laser was mounted on a height adjustable stand and could also be rotated for different measurement angles. The laser was connected to a laptop with LK navigator software. The following settings were sent from the software to the laser: 1ms measurements, data points recorded every 100th measurement, for 65,000 data points. The actuator was positioned using a clamp stand horizontal and perpendicular to the laser beam. The laser was pointed at the actuator and the distance between the device and the actuator

adjusted until the light on the device head flashed green. The device could then be zeroed, and data recording could begin. One cycle lasts 2 minutes – 1 minute heating, 1 minute cooling down. The temperature of the actuator was measured every 5th cycle.

1.7.6 Contact angle measuring

Contact angle measurements were taken on a DSA100 (KRÜSS, UK), with 2 µl of PBS droplets. The measurements were conducted at a temperature of 20 ± 0.5°C. The droplet images were captured across 600 s.

1.7.7 Cell culture

Saos-2 cells (89050205, Merck) were thawed from liquid nitrogen storage and cultured in McCoy's 5A medium (HyCloneTM, UK) with 10% fetal bovine serum (v/v) (Thermo Fisher Scientific, UK), 100 units/mL penicillin (Thermo Fisher Scientific, UK), and 100 µg mL^{-1} streptomycin (Thermo Fisher Scientific, UK), and incubated at 37 °C with 5% CO_2. Once the cells reached 80% confluency, they were washed with Dulbecco's phosphate-buffered saline (DPBS, Thermo Fisher Scientific) and treated with 5 mL of trypsin-EDTA solution (Sigma-Aldrich, UK) for 3 min. The cells were then counted using a haemocytometer before being seeded (5 x 103 cells per well) onto the 24-well plates. Bio35 samples (N = 6) were placed into transwell inserts for 24-well plates (PET, translucent, 3 µm pore size; Sarstedt, Germany) and then added to the 24-well plates seeded with cells. A total of 500 µL of media was added to each well. Also, as a control, Saos-2 cells were seeded directly on 24 well-plates without any samples (no material, N = 6).

1.7.8 alamarBlue Assay

The metabolic activity of Saos-2 cells was assessed using the alamarBlue assay (Thermo Fisher Scientific). The alamarBlue stock solution was mixed with the cell culture medium in a 1:10 ratio to create the alamarBlue/medium solution. On days 1, 7, and 10 of culture, 500 µL of this solution was added to each well and incubated for 2 h. Subsequently, 100 µL from each well was transferred to a 96-well plate in triplicate. Readings were taken using a microplate reader (SYNERGY-H1, BioTek Instruments, Inc.) set to excitation and emission wavelengths of 560 nm and 590 nm, respectively."

For extended cyctoxicity studies: The metabolic activity of Saos-2 cells was assessed using the alamarBlue assay (Thermo Fisher Scientific). The alamarBlue stock solution was mixed with the cell culture medium in a 1:10 ratio to create the alamarBlue/medium solution. On days 1, 7, and 10 of culture, 500 µL of this solution was added to each well and incubated for 2 h. Subsequently, 100 µL from each well was transferred to a 96-well plate in triplicate. Readings were taken using a microplate reader (SYNERGY-H1, BioTek Instruments, Inc.) set to excitation and emission wavelengths of 560 nm and 590 nm, respectively.

For the effect of actuation: A total of 1×10^5 cells were seeded per well onto 24-well plates and cultured for 24 h in an incubator at 37 °C with 5% CO_2. Actuation was then applied to the seeded Saos-2 cells using the Bio35 actuator, powered by an EL 155R power supply, at 3 V (5 and 10 cycles) and 5 V (5 and 10 cycles), with each cycle lasting 1 minute (N = 3). During actuation, the Bio35 actuator was immersed in the well to make contact with the cell monolayer and assess its effect on the cells. Following actuation, cell metabolic activity was evaluated using the alamarBlue assay, as described previously. Seeded cells on tissue culture plastic with no actuation served as the control (N = 3).

1.7.9 Confocal fluorescence microscopy

The Bio35 samples were sterilised with three washes in 70% ethanol for 10 min each, followed by three washes in sterile Dulbecco's phosphate buffered saline (PBS) for 10 min each. The Bio35 samples were then soaked in cell culture media for one day, before seeding Saos-2 cells at a density of 5×10^3 cells

per sample, in 25 µL of cell suspension. The cell-seeded samples were incubated for 1 h at 37 °C with 5% CO_2, before adding 1 mL of cell culture media. Tissue culture plastic (TCP) was seeded in the same way and used as a control. After 24 h of culture, the samples were fixed in 3.7% formaldehyde (Sigma-Aldrich, UK) for 20 min, gently washed with PBS, permeabilised in 0.1% (v/v) Triton X-100 (in PBS, Sigma-Aldrich, UK) for 20 min, then washed three times with PBS. F-actin filaments were stained by incubating with a 4 U mL^{-1} solution of phalloidin conjugate (FITC, Thermo Fisher Scientific, UK) in the dark for 30 min. Samples were washed with PBS, then cell nuclei were stained by incubating with a 1 µg mL-1 solution of 4′,6-diamidino-2-phenylindole (DAPI, Thermo Fisher Scientific, UK) in the dark for 15 min. The samples were then washed with PBS and imaged with a Leica SP8 confocal fluorescence microscope using a 110x HC PL APO CS2 objective. DAPI was imaged using a 405 nm laser excitation and detected in the 415–480 nm range. FITC was imaged using a 488 nm laser excitation and detected in the 498–550 nm range. Z-stack images (1024 × 1024 pixels) were captured for each sample and then combined into a single image using maximum projection in LAS X software.

2 Results

2.1 FTIR analysis

FTIR was used to confirm the degree of polymerization. To avoid the presence of unreacted functional groups in the polymer and prepare a fully crosslinked structure, the crosslinkers, and the prepolymer were maintained in a 1:1 molar ratio. **Figure S1a** show the FTIR spectra of all used reagents with highlighted peaks characteristics for functional groups. The peak at 3200 cm^{-1} of the epoxide group, which is easily recognized in the spectra of the starting material, is missing in all prepared polymer spectra (**Figures S1b-g**). It means all the epoxide groups from the Epikote 828 reacted. Additionally, the crosslinkers with diamine groups exhibited characteristic absorbance peaks in the 3300-3500 cm^{-1} region, which represents asymmetrical and symmetrical stretches of the primary amine end groups, **Figures S1b-g**. In the prepared polymers, these peaks were replaced by a spike in the region from 3300 to 3500 cm^{-1}. It is characteristic of secondary amine stretches, which were created after the reaction between the epoxide ring and the primary amine. A strong absorption peak in the region of 2800 to 3000 cm^{-1} indicates the carbon backbone of the polymer structure, which is displayed in both the reagents and the prepared polymers spectra.

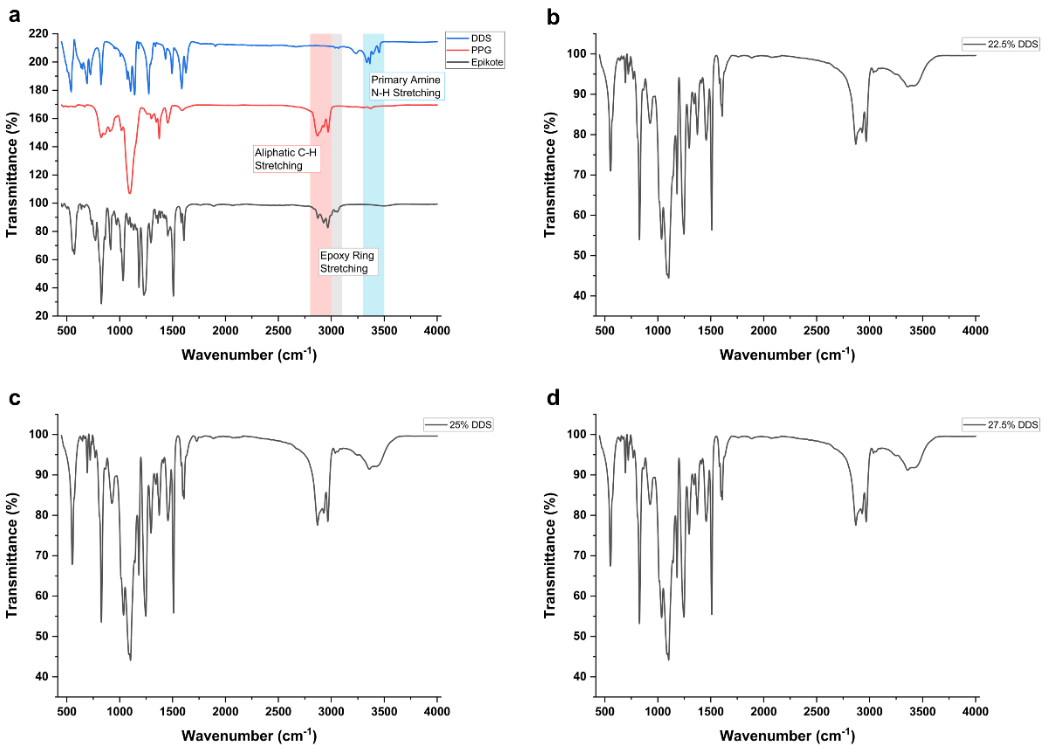

a
DDS
PPG
Epikote
Primary Amine N-H Stretching
Aliphatic C-H Stretching
Epoxy Ring Stretching
Transmittance (%)
Wavenumber (cm-1)
b
22.5% DDS
Transmittance (%)
Wavenumber (cm-1)
c
25% DDS
Transmittance (%)
Wavenumber (cm-1)
d
27.5% DDS
Transmittance (%)
Wavenumber (cm-1)

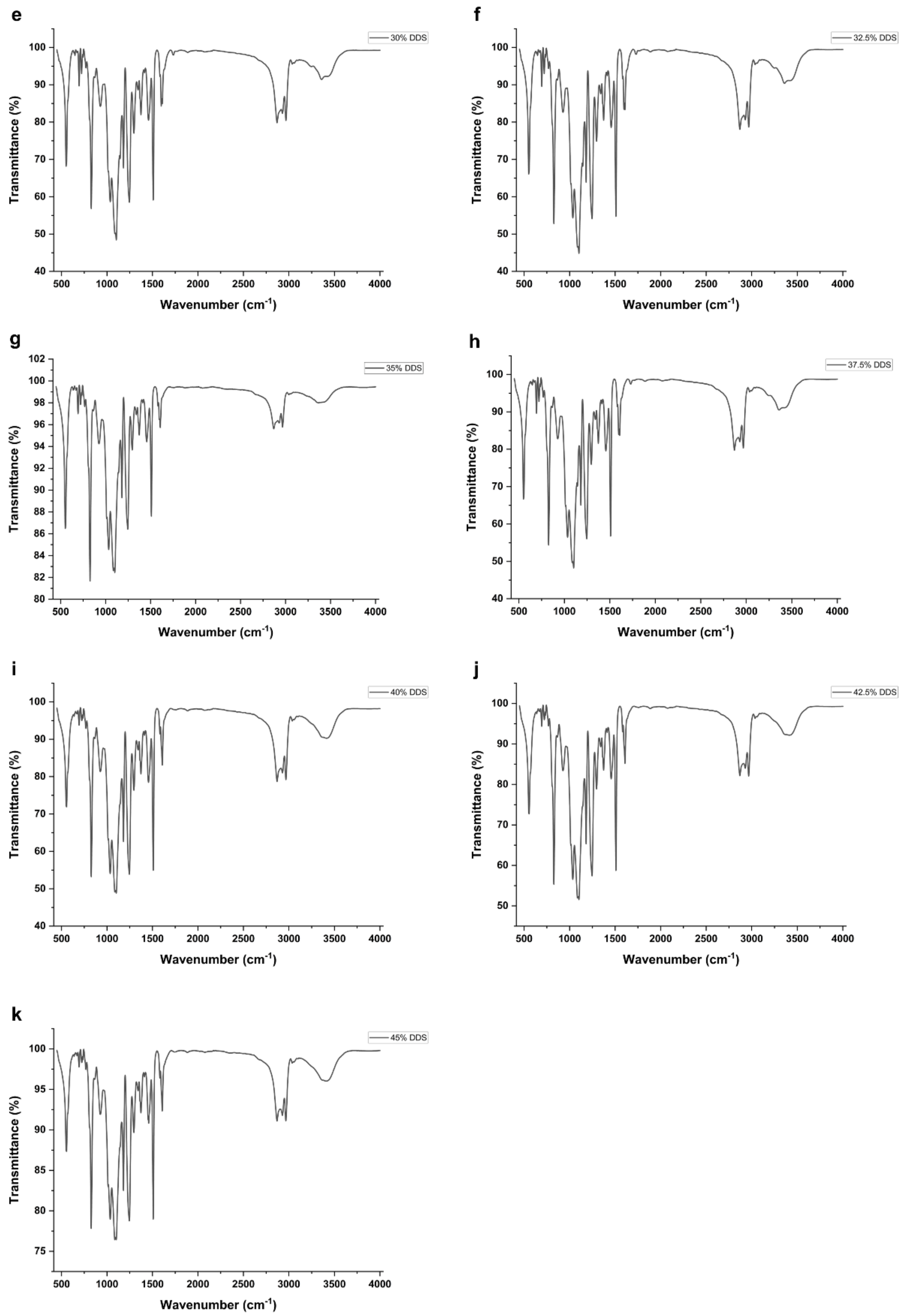

Figure S1. FTIRs where **a)** is starting materials, **b-k**) are actuator epoxy layers with 22.5% DDS to 45% DDS respectively.

2.2 TGA

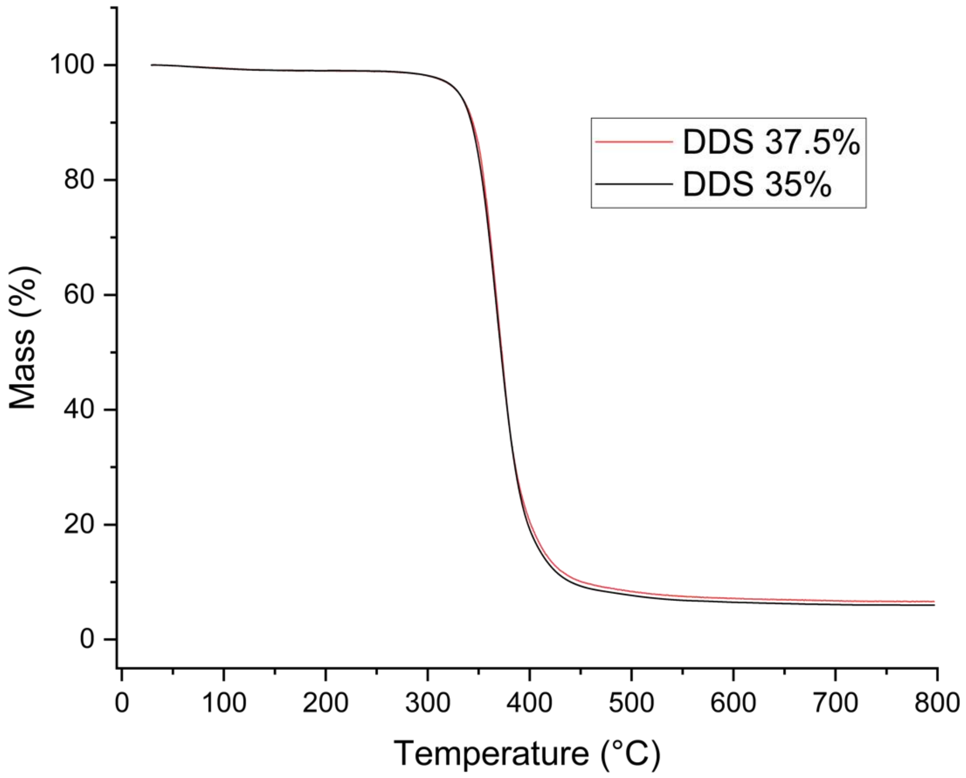

Figure S2. Graph showing thermal stability of prepared actuators (35 and 37.5%) measured by Thermogravimetric analysis

2.3 Gripper actuators

Video S1. Gripper lifting the paper ball (225.1 mg) in atmospheric condition

Video S2. Gripper lifting the paper ball (225.1 mg) in PBS

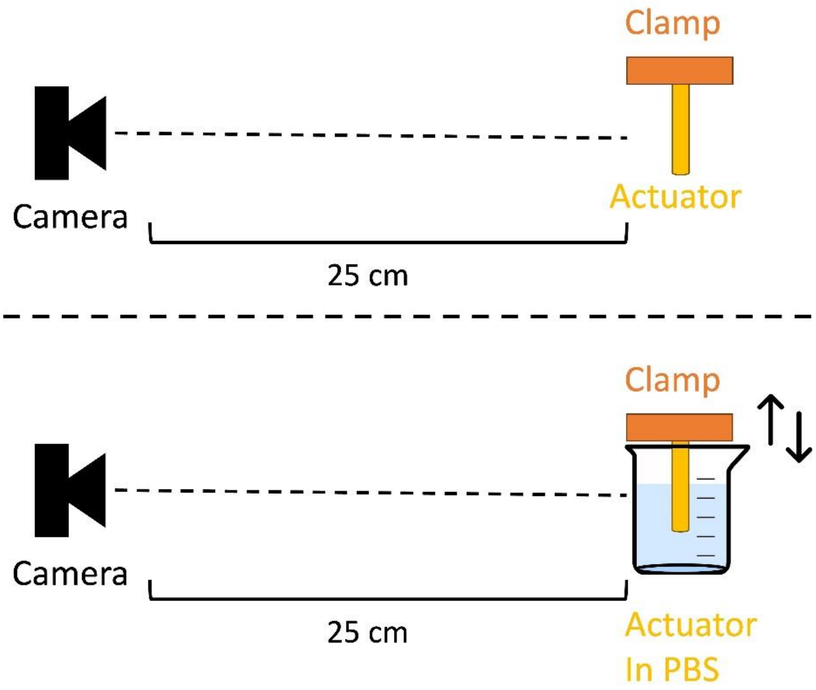

Figure S3. Experimental set up for the images obtained in **figure 1 b** and **c** and **videos S1** and **S2**.

2.4 Force measurements

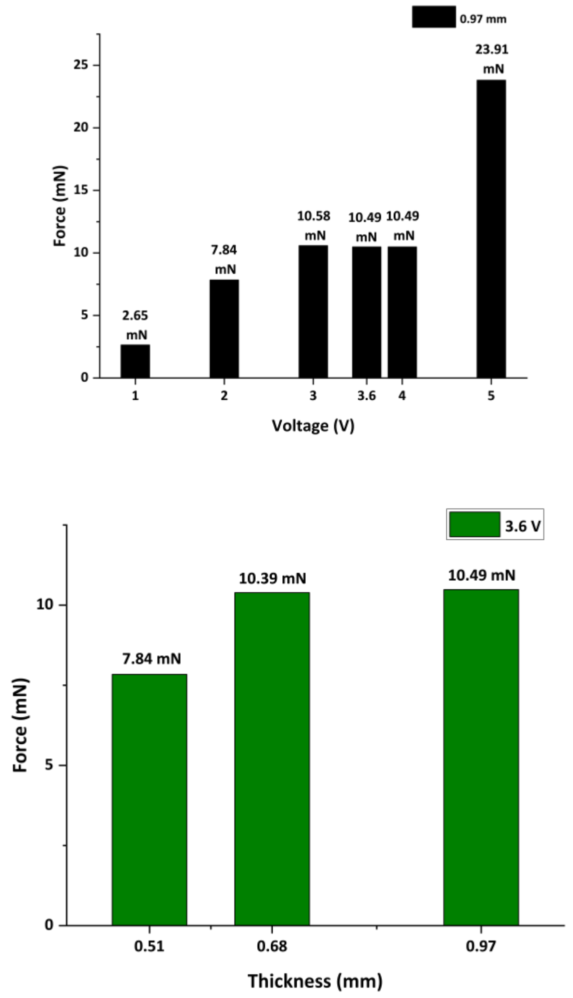

Figure S4. Isometric force generated as a function of **a)** voltage applied on a 0.97 mm thick actuator and **b)** thickness from bilayers of 60 mm length by 12.5 mm width. In each case green bars represent biocompatible actuation temperatures.

2.5 Contact Angle measurements

Table S2: Contact angles of DI water and PBS drops on both layers (resin layer 35 and 37.5%, and Kapton layer)

**sample**		**contact angle 1 [°]**	**contact angle 2 [°]**	**liquid**
35% DDS	resin layer	109.9	111.6	DI water
	resin layer	83.6	83.2	PBS
37.5% DDS	resin layer	93.8	95.3	DI water
	resin layer	95.7	95.4	PBS
Kapton tape		80.1	81.6	DI water
		72.5	73.9	PBS

2.6 Cyclic actuation

To prove the cyclability and durability of the actuator, we measured 100 actuation cycles, see **Figure S4**. It is noted that the actuator can do more than 200 cycles, as we measured for this range the stability of the actuator's temperature. The maximum temperature that it achieved after applying 3.6V was 42.8°C, and the minimum after cooling it down was 21°C. The displacement ranged around the same value of approximately 3.5 mm.

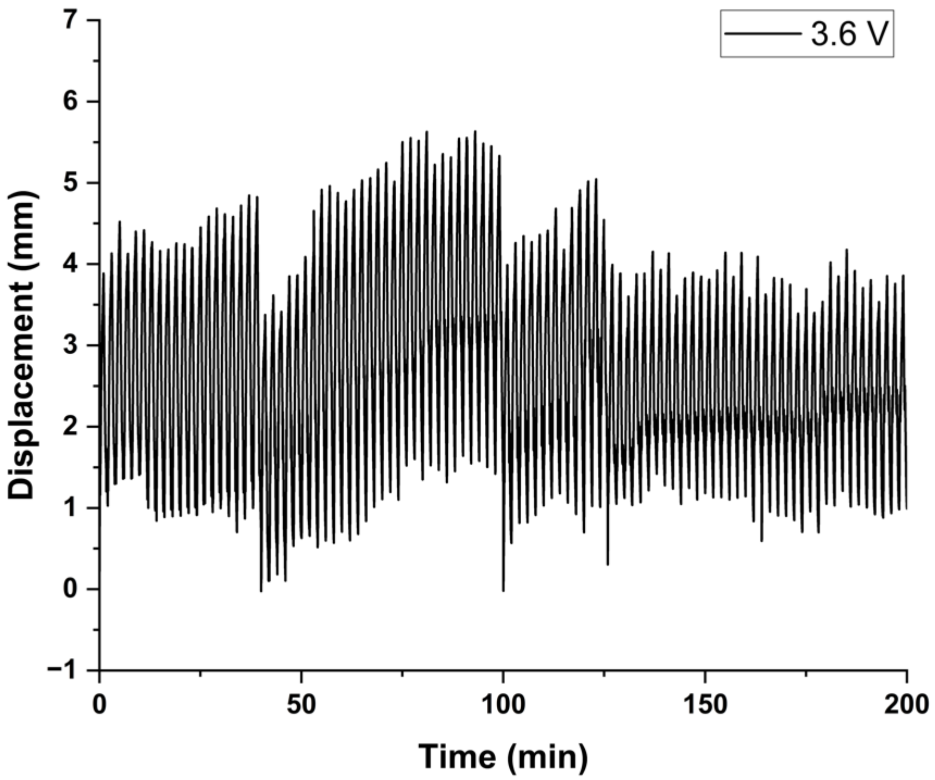

Figure S5: The unnormalized graph of displacement of 100 cycles of the actuator over time. One cycle takes 2 minutes (1 minute heating and 1 minute cooling down). The applied voltage was 3.6V.

2.7 Additional Actuation Data

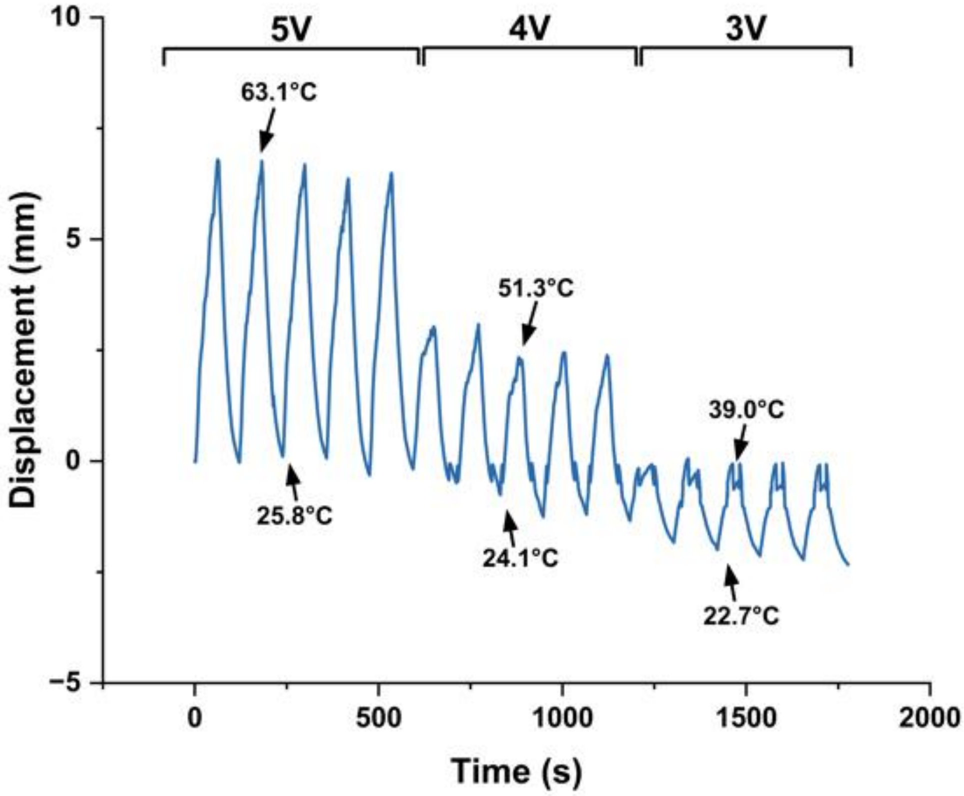

Figure S6. Displacement of actuators cured at 35°C at 5V, 4V and 3V for consecutive cycles.

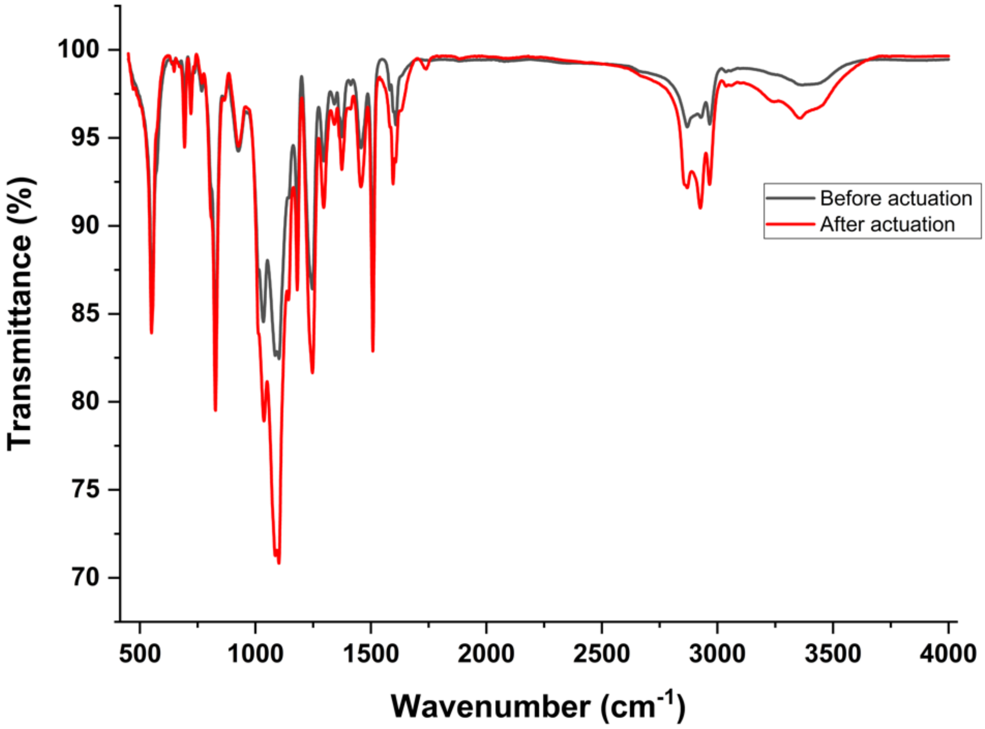

Figure S7. FTIR of actuator before and after repeat cycles.

2.8 Valve additional data

In addition to the data presented in the main manuscript ten consecutive measurements of the rate of flow through a sphincter that has 3.6V applied to it and still has the voltage on (dark blue lines), followed by the flow rate when the sphincter but has only been given a one minute to cool and has no applied voltage (light blue lines) (**Figure S8**).

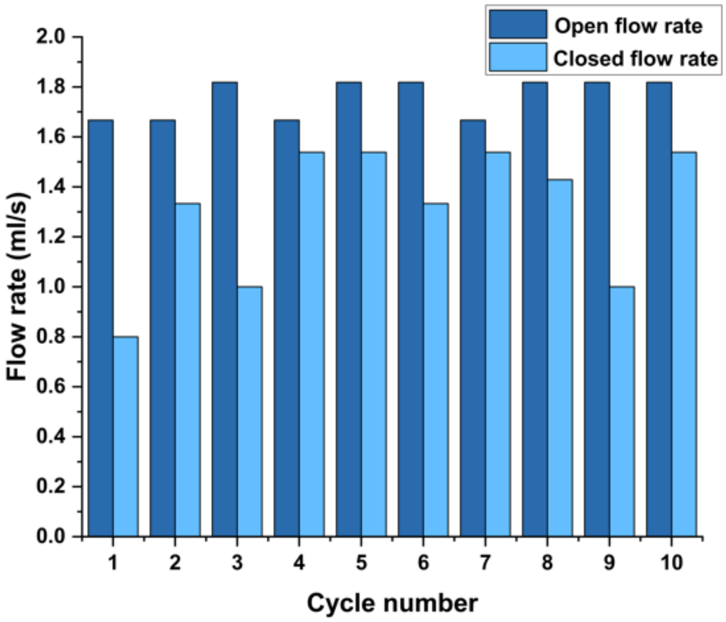

Figure S8. Shows relative flow rate of water through the artificial urethra tubing through the set up in **Figure 7a** when the valve has 3.6 V applied and allowed only 1 minute to call rather than 2.

Video S3. Bladder valve opening (source on) and closing (source off)

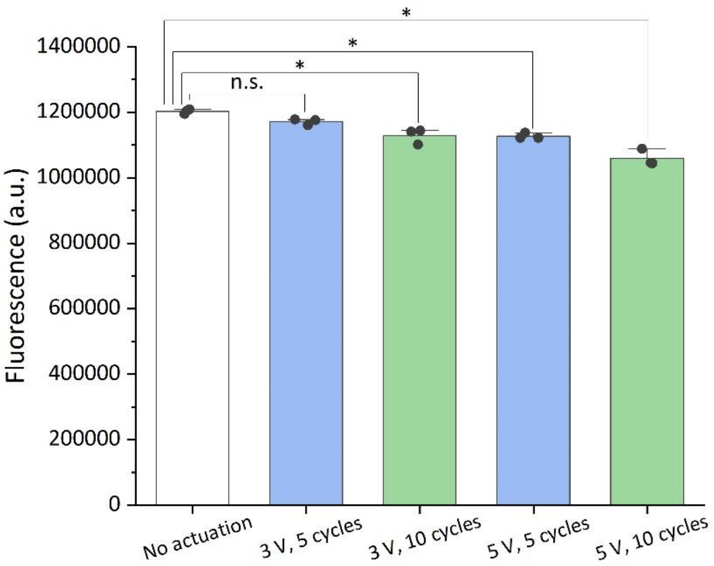

Figure S9. Effect of actuation on Saos-2 cells using two voltages (3 V and 5 V) and two cycle counts (5 and 10). Metabolic activity decreased significantly with increasing voltage and cycle number (* $p < 0.05$).

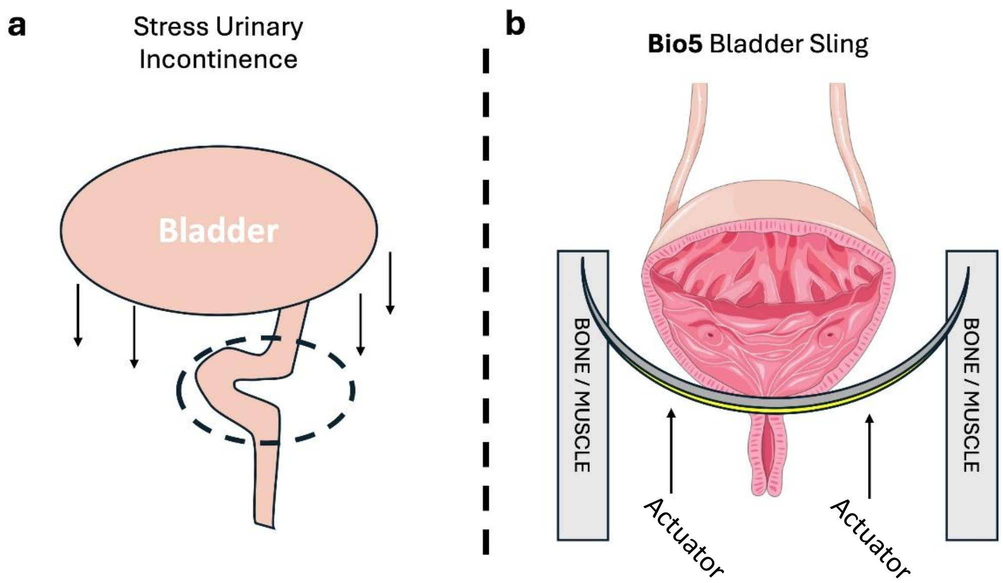

Figure S10. Schematics showing a) where the proposed actuator would be placed to contribute to solutions to stress urinary incontinence; b) the proposed actuator attachment to a sling to give aid to reduce stress urinary incontinence.
