# Supplementary material for: Biocompatible low-voltage electrothermal actuators with biological operational temperature range
Source: Commun Mater. 2025 Aug 5;6(1):174. doi: 10.1038/s43246-025-00893-1 (PMC12325083; doi:10.1038/s43246-025-00893-1)
Supplement: Supplementary file 3 — DOSAF [file 43246_2025_893_MOESM3_ESM.docx]

Description of additional supplementary file

File name: Supplementary Video 1

Description: Actuator video 1 - gripper

File name: Supplementary video 2

Description: Actuator video 2 - submerged

File name: Supplementary video 3

Description: Actuator video 3 – valve

File name: supplementary information

Description: all data sets referred to in main article
